# The relationship between worry and attentional bias to threat cues signalling controllable and uncontrollable dangers

**DOI:** 10.1371/journal.pone.0251350

**Published:** 2021-05-13

**Authors:** Jessie Georgiades, Kelly Cusworth, Colin MacLeod, Lies Notebaert

**Affiliations:** School of Psychological Science, Centre for the Advancement of Research on Emotion, The University of Western Australia, Perth, Western Australia, Australia; University of Pittsburgh, UNITED STATES

## Abstract

People vary in the frequency with which they worry and there is large variation in the degree to which this worry disrupts their everyday functioning. Heightened tendency to experience disruptive worry is characterised by an attentional bias towards threat. While this attentional bias is often considered maladaptive, it can be adaptive when it concerns threat cues signalling dangers that can be mitigated through personal action. In this case, the resulting worry may increase the likelihood of this action being taken, with beneficial rather than disruptive consequences for everyday functioning. Thus, depending on its focus, attentional bias to threat could potentially drive worry that is high or low in disruptiveness. The current study addressed this possibility, by testing the novel hypothesis that the degree to which worry is disruptive is a function of the degree to which this attentional bias concerns all threat cues, rather than being restricted to threat cues signalling controllable dangers. Participants completed a novel probe task assessing their attention to threat cues signalling a future danger that could be controlled on some blocks, but not on others. Thus, the task revealed the degree to which their selective attention to threat cues was ‘aligned’ with danger controllability, by being more evident on blocks that permitted participant control of the danger signalled by the threat cues. The results indicate, contradicting the hypothesis under test, participants who reported high levels of disruptive worry demonstrated alignment of attentional bias to variations in danger controllability, whereas this was not the case for participants who reported high levels of non-disruptive worry. While caution is needed in the interpretation of the results due to methodological limitations, this study provides a new conceptual and methodological framework for future research on the attentional basis of individual differences in the tendency to experience disruptive vs non-disruptive worry.

## Introduction

Most of us strive to attain emotional well-being and to successfully achieve personal objectives (i.e. situational well-being; [1, 2]). However, worry can impact on these goals. Worry involves the mental preoccupation with the aversive implications of a potential future negative event, the probability of the event occurring and the factors that may influence such probability [3]. A heightened disposition to worry excessively is associated with an increased risk of developing clinical anxiety disorders [3, 4] and impaired functioning in social, occupational and academic settings [5]. Therefore, such worry can have a negative impact on emotional well-being [6].

However, worry can also yield benefits as it can operate as a “mental problem-solving activity designed to prevent the occurrence of traumatic future events” [7]. As such, a heightened disposition to worry may at times benefit situational well-being and thus have utility, despite it having a negative impact on emotional well-being.

People vary not only in the degree to which they find their worry to have utility, but also in the degree to which their worry disrupts everyday functioning. Indeed, research has shown that some individuals who experience a high frequency of worry show little evidence that such worry disrupts their everyday functioning, and due to this lack of disruption, such individuals fail to meet criteria for a diagnosis of Generalised Anxiety Disorder [8, 9]. The degree to which this worry disrupts everyday functioning may be related to the controllability of the future negative events people worry about. If an individual worries about a controllable future negative event, worry may be experienced as having utility to situational well-being [10, 11], and this may be perceived to outweigh the costs worry has on emotional well-being. If this is the case, it is reasonable to assume this worry regarding controllable future negative events will be subjectively appraised as being non-disruptive to one’s work, home upkeep and social functioning (i.e., everyday functioning). This is supported by Brandtstadter & Rothermund’s Dual Process Model [12] which suggests that if an individual has control over an outcome, and is able to invest intentional cognitive and behavioural efforts to modify this outcome, this can lead to the attainment of their goal and thus, improve situational well-being.

In contrast, if worry is experienced as detrimental to one’s emotional well-being and has no utility in benefiting situational well-being because the future negative event the individual is worrying about is out of their control [11, 13, 14], it is reasonable to assume that this worry will be subjectively appraised as being disruptive to one’s everyday functioning. This is supported by previous research that has found some individuals who experience a high worry frequency, and meet criteria for a diagnosis of Generalised Anxiety Disorder as such worry disrupts everyday functioning, report frequently worrying about uncontrollable future negative events [13]. Research has also found that worry regarding uncontrollable future negative events comes at a cost to other resources, and the pursuit of other goals including home management, social activities and work activities, and therefore, does not benefit situational well-being [5]. Research has also shown that disruptive worry leads to more negative outcomes as compared to non-disruptive worry [9, 15], including a vulnerability to Generalised Anxiety Disorder [16], which is costly to not only an individual but also society [5, 17]. As such, it is important to understand the mechanisms differentiating these two types of worry. Thus, the current study aims to examine the underlying attentional mechanisms which may differentiate those who experience worry as disruptive, from those who experience worry as non-disruptive.

### Worry and attentional bias

According to the Cognitive Model of Pathological Worry, attentional processes are implicated in individual differences in the tendency to experience worry [18]. Specifically, the model proposes that worry is a function of both top-down (i.e. strategic) processes such as attentional control, and bottom-up (i.e. automatic) processing biases in attention. Previous research has provided support for this model, showing that individuals characterised by a heightened tendency to worry display an attentional bias towards threat [19–21]. This attentional bias reflects increased selective attention to threatening information, compared to benign information in the environment [22]. Studies have demonstrated that individuals who more frequently experience worry, whether this worry is disruptive to everyday functioning (e.g., individuals diagnosed with Generalised Anxiety Disorder), or whether this worry is non-disruptive to everyday functioning, display an elevated attentional bias to threat [23–26]. Research has also established that an attentional bias to threat causally contributes to dispositional worry ([24, 27], see [28] for evidence of a bidirectional relationship).

While such research has established that a higher frequency of worry is associated with a heightened attentional bias to threat, it does not illuminate the possibility as to why in some people this bias to threat cues leads to disruptive worry, whereas in others it leads to non-disruptive worry. Recent theories suggest that worry may be characterised not by a stable attentional bias, but rather by biases that fluctuate across time, context, and threat stimuli [29–31]. Thus, one intriguing potential explanation is that this distinction between those who experience worry as disruptive, from those who experience worry as non-disruptive to everyday functioning, may reflect a difference in the flexible allocation of attention to particular types of threat cues. The current study aims to test whether this is the case.

Several theories suggest that an attentional bias to threat can have an adaptive function, as it allows an organism to prepare for and avoid upcoming dangers [32, 33]. As the ability to fulfil this adaptive function requires the upcoming danger to be controllable, optimal functioning requires increased attentional allocation to threats signalling more controllable dangers and decreased attentional allocation to threats signalling less controllable danger. This pattern of attention allocation is referred to as *attentional bias alignment* [31]. Several studies have now demonstrated that individuals in general indeed flexibly align their attentional bias to such variation in danger controllability, to show a greater attentional bias to threats signalling more controllable dangers relative to threats signalling less controllable dangers [31, 34]. In the current study, we propose that a decreased ability to show such attentional bias alignment contributes to experiencing disruptive worry in particular.

According to Attentional Control Theory [35], worry and attentional processes make use of the same, limited capacity cognitive resources. Increased worry is therefore associated with a heightened reliance on bottom-up attentional processes and a reduced influence of top-down processes, and hence an increased attentional bias to threat. However, the theory also posits that this trade-off between worry and top-down attentional control can be overcome if additional cognitive resources can be recruited [35]. As such, when an individual has sufficient cognitive resources, they should be able to recruit attentional control processes to align their attentional bias with variations in danger controllability. As these individuals will only show an attentional bias to threat cues signalling dangers that can be controlled through personal action, the type of worry that increases the prospect of taking action to mitigate the danger will be activated. This can then reduce or eliminate the danger, which would be beneficial to situational well-being [33], and thus, this worry can be experienced as non-disruptive to everyday functioning. In contrast, when individuals have insufficient cognitive resources to recruit attentional control processes to align their attentional bias to variation in danger controllability, they will attend to threat cues whether or not these signal a controllable danger. The increased attentional bias to threat cues signalling an uncontrollable danger will increase worry without improving situational well-being, leading to worry that is experienced as disruptive. Specifically, the investment of time, energy, and cognitive effort in the worry process will not only be subjectively aversive and functionally futile, but will come at the cost of interfering with the pursuit of other goals [18, 36], plausibly resulting in worry that disrupts everyday functioning.

In the present study, we thus propose that the mechanism that differentiates those who experience worry that has utility and is therefore subjectively experienced as non-disruptive to everyday functioning, from those who experience worry that has no utility and is therefore subjectively experienced as being disruptive, may be such impaired alignment of attentional bias to variations in danger controllability. In addition to Attentional Control Theory [35], several other theoretical models were drawn upon in the formulation of this hypothesis. The proposal that those who experience the type of worry that is most disruptive to situational well-being will show reduced flexibility in their attentional bias is supported by Clark & Beck’s Cognitive Model of Anxiety [14], which suggests that heightened emotional dysfunction is associated with impaired flexibility in the processing of potential threats. Moreover, the association between attention to threat cues signalling controllable dangers and non-disruptive worry is supported by Brandtstadter & Rothermund’s Dual Process Model [12], which is built on Brandtstadter & Renner’s Theory of Assimilative and Accommodative Processes [37]. These models posit that allocation of attentional resources to goals that can be attained leads to the adaptive attainment of the goal [12]. In contrast, sustained cognitive engagement with goals that cannot be attained, because they are out of the individuals’ control is dysfunctional, and comes at a considerable cost to other goals, as cognitive resources are exhausted [12]. This may subsequently lead to further psychological distress [12, 38].

### Current study aims and hypotheses

The aim of the current study was to test the novel hypothesis that, while individual differences in the frequency with which individuals experience worry is a function of the magnitude of attentional bias to threat cues, the degree to which worry is experienced as disruptive is a function of the degree to which there is impaired alignment between attentional bias to threat cues, and the controllability of the danger signalled by these threat cues. Testing this hypothesis will advance our understanding of the attentional processes underpinning disruptive worry and may inform the development of intervention approaches designed to specifically target the cognitive underpinnings of disruptive worry.

To empirically test the validity of the predictions generated by this alignment hypothesis, the current study compared three groups of participants on an Attentional Bias Alignment Assessment Task. One group of participants reported having low worry frequency (Low Worry group), one group reported having high worry frequency that impacted emotional well-being but was not disruptive to situational well-being or everyday functioning (High Worry–Non-Disruptive group), and one group reported having high worry frequency that impacted emotional well-being and was experienced as being disruptive to situational well-being or everyday functioning (High Worry–Disruptive group).

All participants completed an Attentional Bias Alignment Assessment Task, designed to reveal the degree to which they exhibited an attentional bias to threat that was aligned with the degree to which they could control the danger signalled by these threat cues. This task was a novel adaption of the dot probe approach, within which threat cues always signal a future danger, and in some blocks of trials (Control Possible blocks), but not in others (Control Not Possible blocks), participants have the ability to control this danger by performing an adaptive action [31]. Participants’ attentional bias to threat cues is revealed by the degree to which they were relatively speeded to discriminate the identity of probes that appeared in the locus of threat cues, compared to those that appeared in the locus of reward cues [22]. A measure of alignment of attentional bias was then computed for each participant, reflecting the degree to which such attentional bias to threat cues was relatively greater in Control Possible blocks, than in Control Not Possible blocks.

If heightened worry frequency is associated with greater attention to threat, then participants in both the High Worry–Disruptive and High Worry–Non-Disruptive group will display a greater attentional bias to threat cues overall, in comparison to the participants in the Low Worry group. However, the alignment hypothesis under test predicts that participants in the High Worry–Disruptive group will be characterised by greater impaired alignment of attentional bias to threat cues with variations in danger controllability, than will be the case for participants in the High Worry–Non-Disruptive group or in the Low Worry group.

## Method

### Participants

To obtain three groups of participants characterized by differences in worry frequency and worry-related disruption, candidate participants from the University of Western Australia’s School of Psychological Science’s undergraduate participant pool completed the GAD-7 questionnaire to assess worry frequency and the associated negative impact worry has on emotional well-being [39], and an additional question designed to assess the degree to which worry disrupts everyday functioning (worry-related disruption, i.e. situational well-being), as described in the Materials section. The Low Worry group was recruited from the candidate participants who scored low on worry frequency (GAD-7 score ≤ 4). The High Worry-Non-Disruptive group was recruited from the candidate participants who scored high on worry frequency (GAD-7 score ≥ 10) and low on worry-related disruption (worry-related disruption score ≤2). The High Worry–Disruptive group was recruited from the candidate participants who scored high on worry frequency (GAD-7 score ≥ 10) and high on worry-related disruption (worry-related disruption score = 3). As the study involved coloured visual stimuli and auditory stimuli, participants were required to have normal or corrected to normal vision, no colour blindness, or hearing problems.

A power analysis was conducted using G*Power 3 [40], based on previous research on worry-linked attentional bias, in which a medium-sized effect was observed [25]. To achieve sufficient power to detect a medium effect size (*f* = .25) with 90% probability (and a conservatively small .2 correction between repeated measures), a sample size of 87 is required.

As such, to account for potential loss of data, ninety-two students participated in the current study in exchange for partial course credit, including 62 females and 29 males (1 participant did not provide gender or age information), with a mean age of 19.32 (SD = 3.82). Participants gave written informed consent and had the option to terminate the experiment at any time.

### Materials

#### Worry frequency

The Generalised Anxiety Disorder- 7 (GAD-7) questionnaire [39] was used to assess participants’ worry frequency and its associated negative impact on emotional well-being. Scores on this 7-item self-report questionnaire measure range from 0 to 21, with higher scores indicating higher frequency of worry. Individuals who score less than 4 on the GAD-7 have been found to experience minimal levels of worry, and individuals who score greater than 10 on the GAD-7 have been found to experience high levels of worry [39]. The GAD-7 is commonly used to measure worry with well-established internal consistency and test-retest reliability [39, 41]. The GAD-7 also has high convergent validity with the Penn State Worry Questionnaire [42]. In the current study, Cronbach’s alpha was 0.90.

#### Worry-related disruption

An additional question was used to assess the degree to which worry disrupts everyday functioning (i.e. the impact worry has on situational well-being) [39]. This question asked, ‘*If you checked off any problems (in the GAD-7)*, *how difficult have these made it for you to do your work*, *take care of things at home*, *or get along with other people*?’. Participants were asked to answer the question on a 4-point Likert scale, ranging from 0 ‘Not difficult at all’ to 3 ‘Extremely difficult’, with higher scores indicating greater worry-related disruption [39]. A score of 2 or less indicates the individual experiences no difficulty to mild difficulties in functioning at work, home and/or socially, due to their worry. Whereas, a score of 3 indicates the individual experience extreme difficulties in functioning at work, home and/or socially due to their worry [39].

#### Shape stimuli

**Fig 1:**
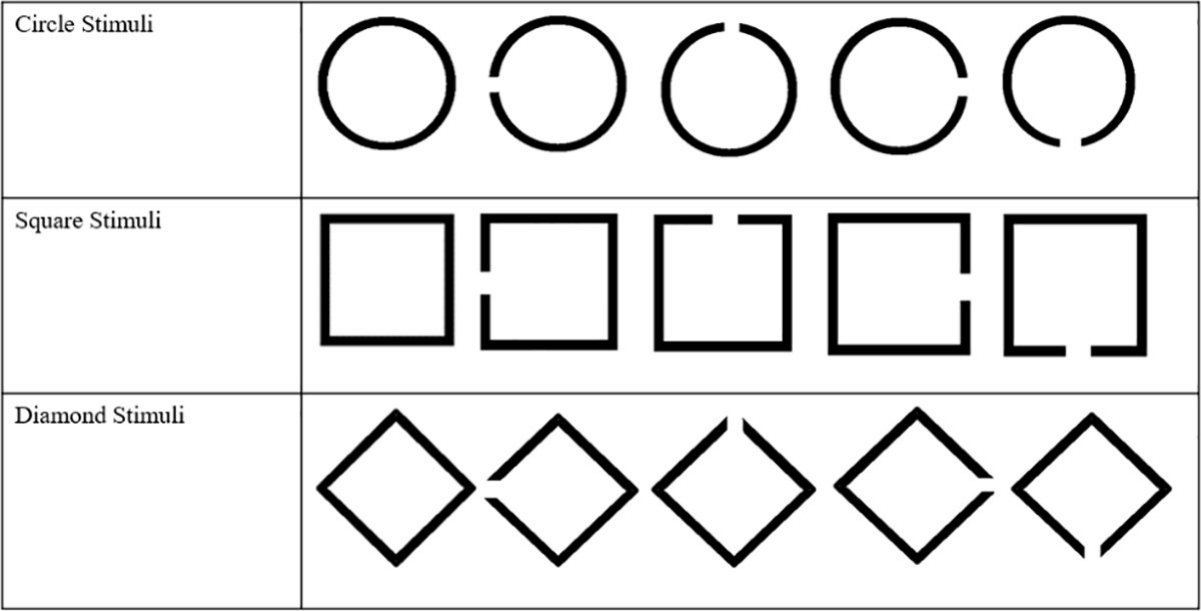
The shape stimuli presented in the Attentional Bias Alignment Assessment Task.

The visual stimuli used in the Attentional Bias Alignment Assessment Task were 3 sets of 5 different 3cm x 3cm images, each representing variants of the same geometric shape. One set contained 5 variants of a circle, one contained 5 variants of a square, and one contained 5 variants of a diamond shape stimuli. Within each set, one shape variant was the complete shape, with no opening in its border outline. The other 4 variants each had an opening in its border outline, which could be an opening on the left side, top, right side, and bottom of the stimuli. Thus, within the circle set, one was a complete shape, whereas the remaining four circles respectively had an opening on the left side, top, right side, and bottom. Within the square set, one was a complete square, whereas the remaining four squares respectively had an opening on the left side, top, right side and bottom. Within the diamond set, one was a complete diamond, whereas the remaining four diamonds respectively had an opening on the left side, top, right side, and bottom. Fig 1 conveys this full set of 15 stimuli.

#### White noise burst

The threat cue used in Attentional Bias Alignment Assessment Task was a shape that signalled a future danger, specifically, a loud noise burst coupled with monetary loss (15 cents) at the end of the trial. The noise burst was a 95-decibel presentation of white noise for a duration of 500ms. Noise bursts delivered for this duration and at this intensity are not physiologically harmful [43], but are subjectively aversive, and previous studies have found evidence of attentional bias towards stimuli that predict such noise bursts [31, 44].

#### Apparatus

The ABAAT was delivered via LG Flatron E2242 27cm x 47cm, computer monitors. The computer was equipped with a standard mouse and keyboard for input of responses. The noise burst stimuli were presented through Logitech z130 speakers and Altec Lansin Speakers, tested to administer the noise burst at 95 decibels. The ABAAT was programmed and presented using the E-Prime 2.0 Software package [45].

### Attentional Bias Alignment Assessment Task (ABAAT)

To examine individual differences in the degree to which attentional bias to threat cues was aligned with variation in the degree to which the danger predicted by the threat cues could be controlled by adaptive action, a new probe-based Attentional Bias Alignment Assessment Task (ABAAT) was developed. This task represents a novel extension of the well-established dot-probe assessment approach [22]. For each participant, one of the three shape stimuli (i.e., circle, diamond and square) was assigned to each of the three following categories: reward cue, threat cue and neutral cue (counterbalanced across participants). Within the task, the *reward cue* signalled the opportunity to gain 5 cents, while the *threat cue* signalled a 50% chance of the danger occurring (i.e., hearing the 95dB noise burst and losing 15 cents). The *neutral cue* signalled neither reward nor danger.

On each trial, two shapes were simultaneously presented for 500ms, one to the left and one to the right of the screen centre. The task was designed such that participants could pursue the goal of gaining money on every trial, although on some trials the presence of a threat cue threatened this goal. As such, in each pair of stimuli, one of the presented shapes was always the reward cue, while the other was either the neutral cue or the threat cue. Trials in which the reward-threat pair were presented were critical in the assessment of participants’ attentional bias towards threat cues relative to reward cues. Trials presenting reward-neutral pairs were included to ensure that threat cues were not consistently presented, thereby ensuring that the presence of the threat cue had predictive value in signalling the future aversive experience of monetary loss and the presentation of the noise burst [31, 46].

Following the presentation of the shape pair, a small probe stimulus was presented in the former location of one of the shape stimuli, and participants were required to swiftly discriminate its identity, and indicate this by quickly pressing one of two response buttons. The distribution of attention between the two stimuli in presented pairs was inferred from the relative latency to discriminate the identity of the probes presented in the former location of each of the two shape stimuli [22, 47]. On trials that presented a reward and a threat cue, an index of attentional bias to threat was provided by computing the degree to which probes subsequently presented in the location of the threat cue were discriminated faster than probes subsequently presented in the location of the reward cue [22, 47].

The task was configured such that attention to the threat cue would be helpful to the goal of earning money in some blocks, however, would interfere with this goal in other blocks. As such, two types of trial blocks were presented that differed in whether or not the danger predicted by the threat cues could be controlled by the participant. This enabled assessment of the degree to which attentional bias to threat was aligned with variations in the controllability of danger. In one type of block (Control Possible blocks) participants were informed that they could eliminate the probability of the danger signalled by the threat cue, by quickly indicating at the end of the trial the location of the opening in the threat cue presented (left, top, right or bottom). In the other type of block (Control Not Possible blocks), participants were informed that they could not control the danger signalled by the threat cues. This danger controllability manipulation provided a measure of alignment of attentional bias to variations in danger controllability. Specifically, alignment of attentional bias would be displayed if participants exhibit a greater attentional bias to threat in Control Possible blocks, than in Control Not Possible blocks.

**Fig 2:**
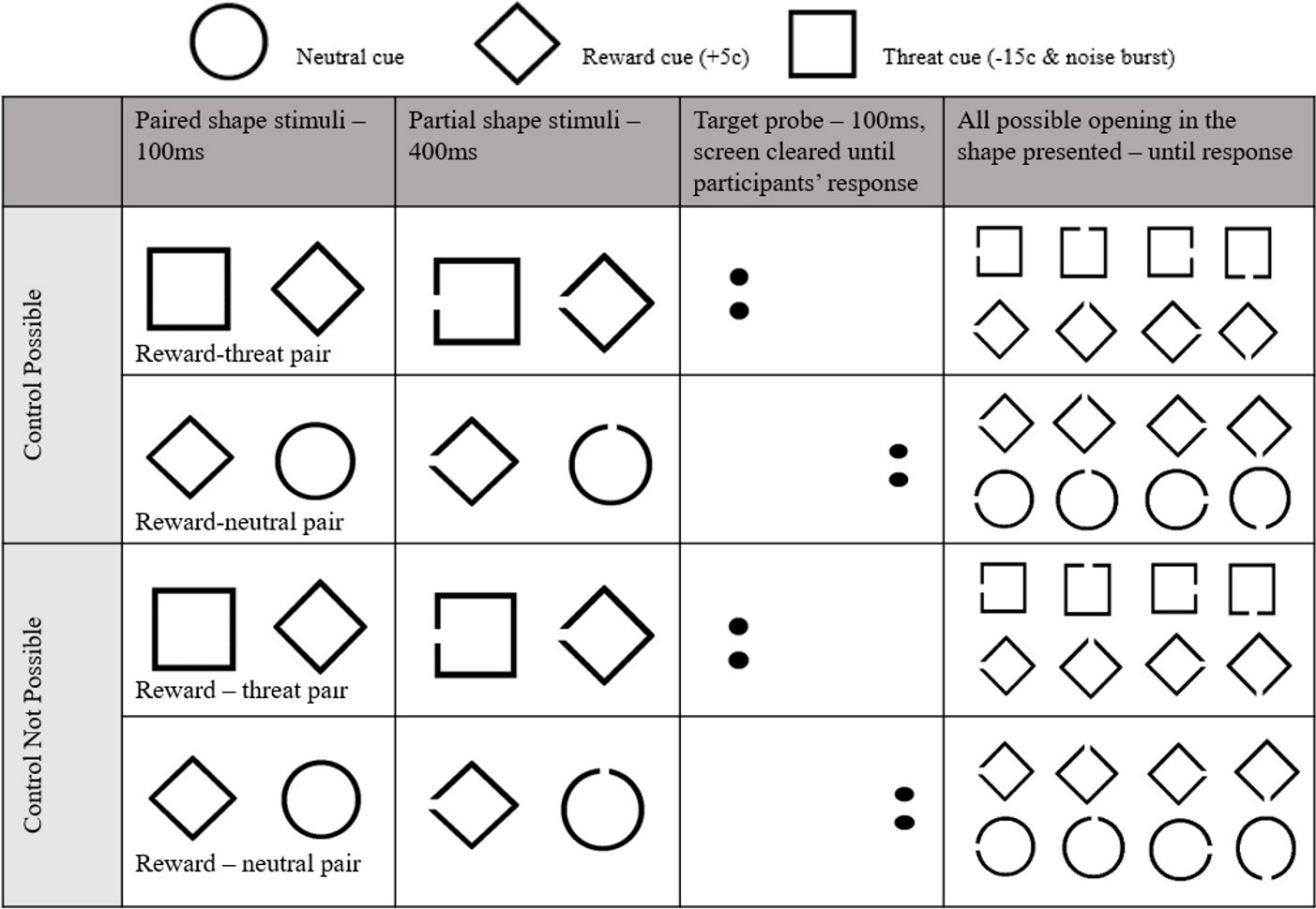
Example trials from the Attentional Bias Alignment Assessment Task.

The detailed nature of each individual trial, summarized in Fig 2, was as follows. On every trial, the shape pair was shown in black, on a grey background, with the centres of the pair stimuli separated by 9cms. The shape pair was presented for 500ms. The two complete shapes were shown for the first 100ms, before these were each replaced by one of the four versions of this same shape with an opening in one of its four sides for the remaining 400ms. Following, the screen was cleared, and a small probe appeared in the former location of either shape stimuli for 100ms. Each probe was a pair of dots, oriented either horizontally (..) or vertically (:) with equal probability, presented in black Calibri script point size 16 on the grey screen background. On half of the trials in each block, this probe appeared in the location previously occupied by the reward cue, and on the other half of these trials the probe appeared in the location previously occupied by the other cue, with location of the probe randomized across trials. Participants were required to accurately identify the probe orientation and indicate this as quickly as possible by using their dominant hand to press ‘1’ on the keyboard if the probe was two dots oriented vertically (:), or ‘2’ on the keyboard if the probe was two dots oriented horizontally (..). The accuracy and latency of participants’ responses were recorded on each trial.

After participants completed the probe discrimination response, irrespective of the accuracy of response, the screen displayed all four variants of the two shapes shown earlier in that trial, each with an opening in one of their sides. The participant was required to click on only one of these shapes. If the participant clicked on the version of the reward cue that had been shown earlier on this trial (i.e., the version with the opening in the same location), then they earned 5 cents. If this reward cue had been paired with the neutral cue in the initially presented shape pair, and the participants clicked on any variant of this neutral cue, when this later screen of 8 stimuli were presented, then this had no consequence. However, if the reward cue had been paired with the threat cue in the initially presented pair, and the participants clicked on the version of the threat cue that had been shown earlier on this trial (i.e., the version with the opening in the same location), then the consequence depended upon whether or not the trial was within a Control Possible block, or a Control Not Possible block. In both block types, when a threat cue had been presented in a trial, then at the point when the participant clicked only one of the 8 final shapes there was a 50% possibility that the noise burst would be delivered, and the participant would lose 15 cents. On Control Not Possible blocks this probability was unaffected by whichever shape variant the participant selected within the final set of 8 shapes. On Control Possible blocks, however, the possibility of the danger occurring was reduced to zero if the participant selected the shape variant corresponding to the threat cue that had been presented in the initial shape pair, when given the final set of 8 shapes.

Thus, in Control Possible blocks, correctly identifying the opening in the threat cue enabled participants to successfully avoid the danger (15-cent monetary loss and noise burst) and therefore, the benefits of which outweighed the monetary reward (5-cents) that could have been gained by instead correctly identifying the opening in the reward cue. In contrast, in Control Not Possible blocks, correctly identifying the opening of the threat cue meant foregoing the 5-cents that would have been gained by instead correctly identifying the opening in the reward cue, without serving to reduce the danger signalled by the threat cue. As such, although the presence of the threat cue consistently predicted the danger, correctly identifying the opening in the reward cue in Control Not Possible blocks gained the participant 5-cents, and thus reduced the magnitude of the 15-cent monetary loss signalled by the threat cue (total loss of 10-cents).

The screen was cleared when participants selected one of the 8 shapes, and the trial ended with a screen showing whether participants had gained money (e.g., “+5”), had lost money (e.g., “-15”), and/or had avoided the danger (e.g. “Noise burst avoided and +0”). After a 1000ms inter-trial-interval, the next trial then commenced. Participants completed four blocks of 64 trials (32 containing reward-threat pairs and 32 containing reward-neutral pairs), two delivered in Control Possible condition and two delivered in the Control Not Possible condition, with the order of these two conditions alternating across blocks, and with the starting condition counterbalanced across participants. Prior to the beginning of each block, participants were explicitly informed whether the block was a Control Possible block or a Control Not Possible block. Following completion of each block, participants were notified of how much money they had earned in that block.

For each participant, an index of attentional bias to threat was first computed for each of these two block types, using those trials in which the threat cue had been presented. This index of attentional bias to threat was calculated by subtracting the identification latency for probes presented in the former location of the threat cue, from the identification latency for probes presented in the former location of the reward cue. Higher scores on this index reflected relative speeding to identify probes in the location of the threat cues compared to probes in the location of reward cues, and thus, indicated greater attentional bias to threat.

The degree to which participants exhibited alignment of attentional bias to threat with variations in danger controllability, will be revealed by the degree to which the above-described index of attentional bias to threat is elevated in Control Possible blocks, relative to Control Not Possible blocks.

### Procedure

Participants were tested individually in sound-attenuated cubicles, in a quiet laboratory setting. Participants first provided demographic information and were then provided with the instructions for the Attentional Bias Alignment Assessment Task. It was also emphasised that they could earn real money in this task. The task started with a staged practice component. The first stage consisted of 12 trials and introduced participants to the shape stimuli (without the gap in one of its sides) and dot probe stimuli. Participants were required to identify the orientation of the probe. Stage 2 consisted of 16 trials and introduced the contingency between one of the shapes and the reward (rendering this shape the reward cue), and participants were informed that the correct identification of the opening in this particular shape at the end a trial would earn them 5 cents per trial. Thus, in this practice stage trials now included the final presentation of all eight possible shape variants. Stage 3 consisted of 16 trials and introduced the contingency between one of the shapes and the danger (rendering this shape the threat cue). During this practice stage, if the threat cue was presented, participants also had a 50% chance of losing 15 cents and hearing the noise burst. However, they were informed that if they correctly identified the reward shape variant on such trials, they still gained 5 cents. Stage 4 consisted of 16 trials and introduced the concept of danger controllability. During this stage, participants were informed that if they correctly identify the position of the opening of the shape deemed the threat cue, then they would successfully avoid the danger (i.e., monetary loss and noise burst). Participants were informed that if they correctly identify the position of the opening of the shape deemed the reward cue, they will still gain 5 cents per trial but would not reduce the probability of the danger (i.e., monetary loss and noise burst) when a threat cue had been presented.

Following the practice trials, participants were notified that in some blocks of the main task, if they correctly identified the position of the opening of threat cues, they could prevent the danger (money loss and noise burst) from occurring, whereas on others this would not be the case. They were advised that they would be told which type of block they were completing at each stage of the task. Participants then completed the four blocks of the attentional bias alignment assessment task. Following completion of each block, participants were notified of how much money they had earned. At the end of the session, the experimenter provided the total amount earned across the four blocks, and participants were debriefed. The study was approved by the University of Western Australia’s Human Research Ethics Committee (RA/4/1/5295).

## Results

All subsequent analyses were conducted using IBM SPSS Statistics for Windows, Version 24.0 [48]. The significance threshold was set at an alpha = .05. For all subsequent analyses, guidelines for F tests suggest a small effect as *η*_*p*_^2^ > .02, a medium effect as *η*_*p*_^2^ > .06 and a large effect as *η*_*p*_^2^ > .14 [49]. For t-tests, guidelines suggest a small effect is equal to *d* > .20, a medium effect as *d* > .50 and a large effect as *d* > .80 [50].

### Data preparation

Prior to data analysis, trials in which participants made incorrect probe identification responses or exhibited outlier identification latencies were removed. Outliers were defined as probe discrimination latencies that deviated more than 2.5 absolute deviations from an individual’s median probe discrimination latency for that trial type (reward-neutral and reward-threat trials), within each of the four Attentional Bias Alignment Assessment Task blocks separately. This resulted in 7.34% of trials being removed from the analyses.

Poor accuracy discriminating the identity of the probes, or identifying the opening of the shape stimuli, would indicate that participants did not comply with task instructions. Thus, it was planned to exclude any participants who exhibited less than 70% accuracy on either of these required decisions [51]. Overall, the probe discrimination accuracy was high (*M* = .98, *SD* = .02) and no participants met this exclusion criterion. However, although accuracy identifying the opening of the shape was also generally high (*M* = .97, *SD =* .05*)*, one participant demonstrated accuracy of only 60.16% and was excluded from the analyses.

The remaining data was subjected to normality testing. Attentional Bias to Threat in Control Not Possible blocks (*M* = -58.16, *SD =* 96.95) identified one participant outlier, with an interquartile range greater than 3 standard deviations from the median of the sample. This participant was excluded from the analyses. Following the data preparation, all variables were normally distributed with a skew < 2 and kurtosis < 4 [52].

### Participant characteristics

The final sample (N = 90) consisted of 29 males, 60 females (1 participant did not provide gender information). The sample consisted of participants of Australian (62%), English (8%), Singaporean (3%), Indian (2%) and other (25%) nationality. Descriptive statistics for age, gender and money earned in the Attentional Bias Alignment Assessment Task, based on groups, are presented in Table 1. There were no significant differences between groups on demographic measures or the amount of money earned, all p > .05.

**Table 1:** Age, gender distribution, and money earned in the Attentional Bias Alignment Assessment Task (M, SD) for the three participant groups.

	Low Worry (*N* = 32)	High Worry—Non-Disruptive (*N* = 30)	High Worry–Disruptive (*N =* 28)
Age	19.84 (4.79)	18.52 (.99)	19.50 (4.45)
Gender (F/M)	18/14	21/9	21/7
Money earned (cents)	402.30 (58.06)	380.00 (87.16)	393.93 (89.35)

### Analysis of Attentional Bias to Threat Index scores in reward- threat trials

**Fig 3:**
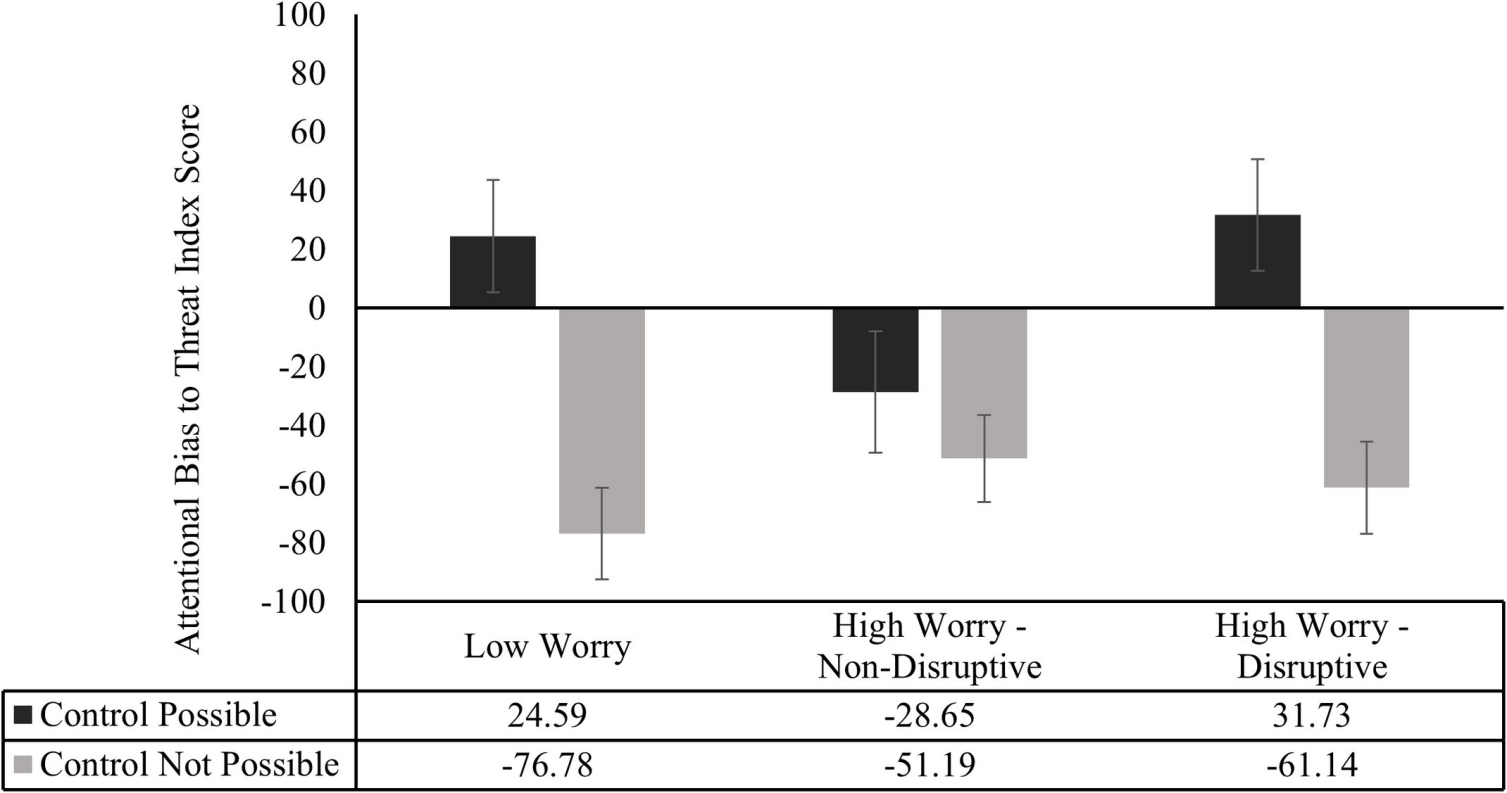
Attentional Bias to Threat Index scores for each group in control possible and control not possible blocks. Errors bars are standard error bars.

To test the predictions generated by the proposed hypothesis, attentional bias to threat index scores evidenced by the three participant groups, in each of the two block types were subjected to a 2 x 3 mixed Design ANOVA (See Fig 3). The within subjects factor was Block Type (Control Possible vs Control Not Possible) and the between subjects factor was Worry Group (Low Worry vs High Worry—Non-Disruptive vs High Worry—Disruptive).

Results revealed a significant main effect of Block Type, *F*(1, 87) = 22.84, *p* = < .001, *Ƞp*^2^ = .21, reflecting that on average participants evidenced higher attentional bias to threat index scores in Control Possible blocks (*M* = 9.07, *SD* = 109.96) than was the case in Control Not Possible blocks (*M* = -63.68, *SD* = 84.13). This finding suggests that averaging across participants, there was evidence that attentional bias to threat cues was aligned with the controllability of the dangers signalled by these threat cues. Contrary to our prediction that the two groups of high worriers would show greater attentional bias to threat than would the low worriers, there was no significant main effect of Worry Group, *F* (2, 87) = 1.09, *p* = .34. However, consistent with the hypothesis’ prediction that attentional bias alignment would differ across the participant groups, the interaction between Block Type and Worry Group trended towards significance, *F* (2, 87) = 2.76, *p* = .07, *Ƞp*^2^ = .06.

As there was a priori hypothesis regarding the nature of this trending interaction, paired samples t-tests (two-tailed) were employed to test whether the index of attentional bias to threat was greater in Control Possible blocks, relative to Control Not Possible blocks, for each Worry Group (See Fig 3). The participants in the Low Worry group displayed significantly higher attentional bias to threat scores in Control Possible blocks, than in Control Not Possible blocks (*M* = 24.59, *SD* = 108.40 vs *M* = -76.78, *SD* = 88.48; *t* (31) = 3.97, *p* < .001, *d* = 0.70). Participants in the High Worry—Disruptive group also displayed significantly higher attentional bias to threat scores in Control Possible blocks, than in Control Not Possible blocks (*M* = 31.73, *SD* = 100.94 vs *M* = -61.14, *SD* = 83.16; *t* (27) = 3.51, p = .002, *d* = 0.66). However, participants in the High Worry–Non-Disruptive group displayed no such difference in attentional bias to threat scores in Control Possible blocks than in Control Not Possible blocks (*M* = -28.65, SD = 113.26 vs *M* = -51.19, *SD* = 80.96; *t* (29) = .85, *p* = .40).

To further assess whether there is a difference in attentional bias to threat in Control Not Possible blocks across the Worry Groups, a One-Way ANOVA was conducted. Results indicated there was no statistically significant difference in the pattern of attentional bias to threat in Control Not Possible blocks amongst the groups, *F* (2, 87) = .73, p = .49. A One Sample t-test (two-tailed) comparing participants’ index of attentional bias to threat in Control Not Possible blocks (*M* = -63.38, *SD* = 84.13) to zero, showed a significant effect, *t* (89) = 7.15, p < .001, *d* = .75, indicating that overall participants displayed an attentional avoidance to threat cues in Control Not Possible blocks.

To further assess whether there is a difference in attentional bias to threat in Control Possible blocks across the Worry Groups, a One-Way ANOVA was conducted. Results indicated a marginal difference in the pattern of attentional bias to threat in Control Possible blocks amongst the groups, *F* (2, 89) = 2.79, *p* = .07, *Ƞp*^2^ = .06. Specifically, participants in the Low Worry and High Worry–Disruptive groups displayed positive scores (*M* = 24.60, *SD* = 108.40, and *M* = 31.73, *SD* = 100.94, respectively), suggesting that such participants displayed greater attention to the threat cue in Control Possible blocks. In comparison, participants in the High Worry–Non-Disruptive group displayed negative scores (*M* = -28.65, *SD* = 113.26), suggesting that participants in this group displayed greater attention to the reward cue in Control Possible blocks.

The addition of Block Order (Control Possible First vs Control Not Possible First) as a factor to this ANOVA did not produce a significant three-way interaction between Block Type, Worry Group and Block Order, *F*(2, 84) = .24, *p* = .79. This result indicates that the observed pattern of results is not different between participants who started with a Control Possible vs a Control Not Possible block. Additional results assessing participants’ response patterns to the shape identification component of the task are presented in S1 File.

Overall, these results indicate that participants in the High Worry—Non-Disruptive group displayed impaired alignment of attentional bias with variations in danger controllability, as they showed greater attention allocation to the reward cue in both block types. In contrast, the other two groups showed evidence of alignment of attentional bias with variations in danger controllability, showing an attentional bias towards the threat cue in Control Possible Blocks, and greater attention allocation to the reward cue in Control Not Possible blocks.

### Attentional Bias to Reward Index scores in reward-neutral trials

Additionally, to explore the pattern of attentional bias to reward cues on reward-neutral trials, an Attentional Bias to Reward Index score was calculated. This Attentional Bias to Reward Index was calculated for reward-neutral trials in each block type, by subtracting the identification latency for probes in the former location of the reward cue, from the identification latency for probes presented in the former location of the neutral cue. Higher scores indicate a greater attentional bias to reward cues.

**Fig 4:**
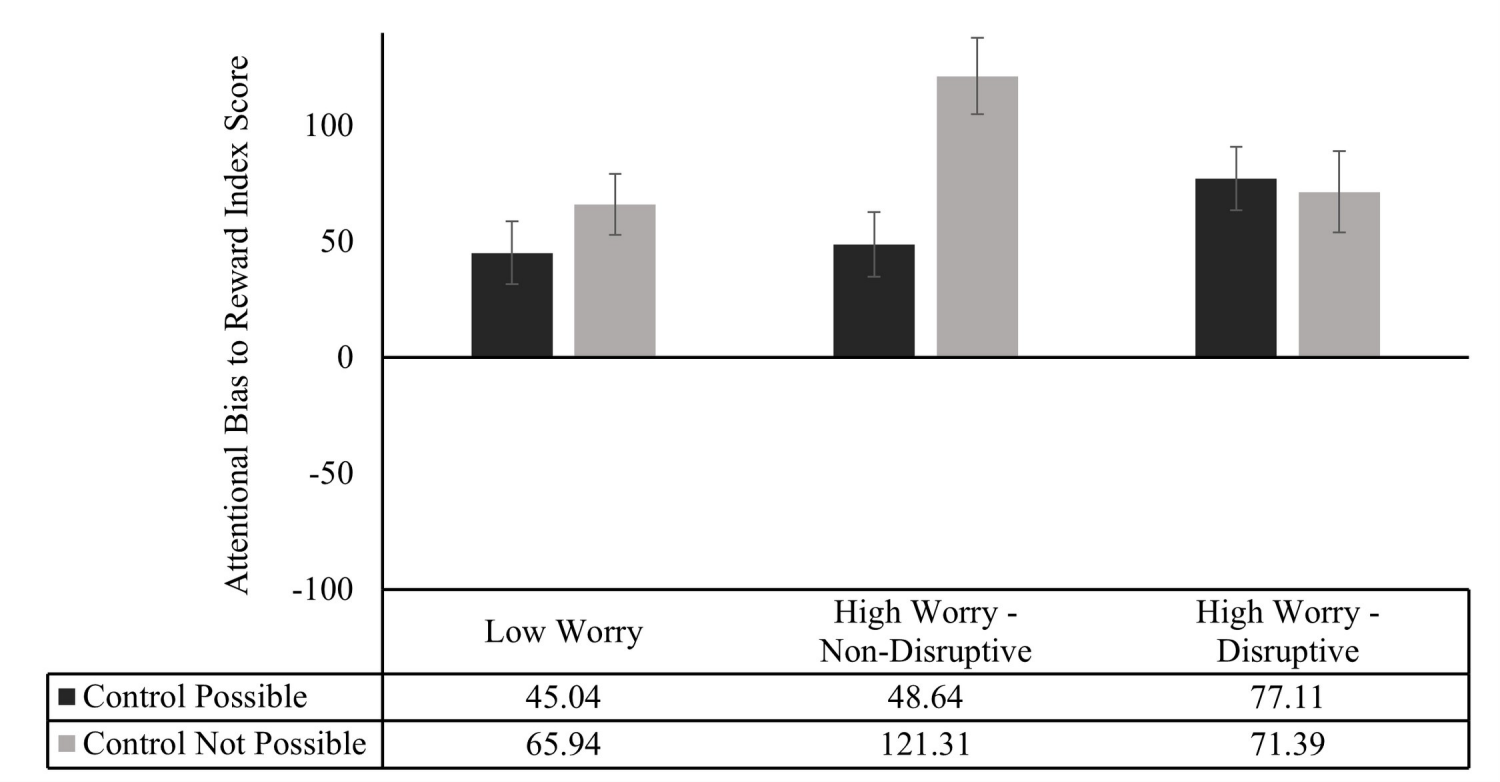
Attentional Bias to Reward Index scores for each group in control possible and control not possible blocks. Errors bars are standard error bars.

To assess participants’ pattern of attention in reward-neutral trials, the Attentional Bias to Reward Index scores evidenced by the three participant groups, in each of the two block types were subjected to a 2 x 3 mixed design ANOVA, using the same approach used previously (See Fig 4). Results revealed a significant main effect of Block Type, *F*(1, 87) = 7.40, *p* = .008, *Ƞp*^2^ = .08, reflecting that on average participants evidenced higher Attentional Bias to Reward Index scores in Control Not Possible blocks (*M* = 86.09, *SD* = 88.08), than was the case in Control Possible blocks (*M* = 56.22, *SD* = 75.42). There was no significant main effect of Worry Group, *F* (2, 87) = 1.80, *p* = .17. The interaction between Block Type and Worry Group was significant, *F* (2, 87) = 4.47, *p* = .01, *Ƞp*^2^ = .09.

To illuminate the nature of this interaction, paired samples t-tests (two-tailed) were employed to test whether the index of attentional bias to reward was greater in Control Not Possible blocks, relative to Control Possible blocks, for each Worry Group (See Fig 4). The participants in the Low Worry group displayed no significant differences in attentional bias to reward in Control Not Possible blocks, than in Control Possible blocks (*M* = 65.94, *SD* = 74.34 vs *M* = 45.04, *SD* = 76.51; *t* (31) = 1.12, *p* = 27). Participants in the High Worry—Disruptive group also displayed no significant differences in attentional bias to reward in Control Not Possible blocks, than in Control Possible blocks (*M* = 71.39, *SD* = 92.15 vs *M* = 77.11, *SD* = 71.79; *t* (27) = .28, p = .78). However, participants in the High Worry–Non-Disruptive group displayed a significantly higher attentional bias to reward in Control Not Possible blocks, than in Control Possible blocks (*M* = 121.31, *SD* = 89.76 vs *M* = 48.64, SD = 76.06; *t* (29) = 4.42, *p* < .001, *d* = -.81).

A series of One Sample t-tests (two-tailed) comparing participants’ Attentional Bias to Reward Index scores in Control Possible and Control Not Possible blocks to zero, for the Low Worry, High Worry—Non-Disruptive and High Worry–Disruptive groups, showed significant effects (all p < .001). This indicates that participants attended to the reward cue in both the Control Possible and Control Not Possible blocks.

The addition of Block Order as a factor to this ANOVA did not produce a significant three-way interaction between Block Type, Worry Group and Block Order, F(2, 84) = .94, p = .39.

Overall, these results indicate that participants in the Low Worry, High Worry-Non-Disruptive and High Worry-Disruptive groups all displayed an attentional bias to reward, in both Control Possible and Control Not Possible blocks, in reward-neutral trials. However, the High Worry-Non-Disruptive group displayed a significantly higher attentional bias to reward in Control Not Possible blocks, than in Control Possible blocks.

## Discussion

The aim of the current study was to test the hypothesis that while elevated worry frequency is a function of the greater attentional bias to threat cues, the degree to which worry is experienced as disruptive reflects differences in alignment between attentional bias to threat cues and the controllability of the danger signalled by these threat cues. Testing this hypothesis was important to advance the understanding of the cognitive processes differentiating disruptive from non-disruptive worry. Results did not support the predictions generated by this hypothesis; however, the observed findings generate a range of questions which are important to address in future research.

We found no evidence that participants in the two groups characterised by elevated worry frequency (i.e., High Worry-Disruptive and High Worry-Non-Disruptive groups) exhibited greater attentional bias to threat cues, than participants in the Low Worry group. This is inconsistent with previous research, which have reported an association between worry frequency and attentional bias to threat [19–21]. Differences in the methodological approaches used in the current study, in comparison to previous studies may account for this discrepancy. Firstly, this discrepancy could be attributed to the type of threat cue stimulus used. Specifically, in the current study the threat cue stimulus that was presented was non-aversive and predicted an upcoming aversive danger. However, previous research assessing worry-linked attentional bias have used threat cue stimuli that has been deemed aversive [53], including threatening IAPs images [54] and threatening words [22]. Thus, it is possible that the association between worry and attentional bias to threat is restricted to aversive stimuli that may be more directly relevant to an individual’s current worries. Secondly, this discrepancy could be attributed to the pairing of threat cues with reward cues in the current paradigm, in contrast to previous studies in which the benign stimuli paired with threat has no functional value to participants [19–21]. In the current paradigm, attending to the reward cue permits the participant to earn money. This was important as the presence of this reward cue rendered attentional allocation to the threat cue in the Control Not Possible blocks objectively maladaptive. However, by encouraging attentional processing of the reward, this may have attenuated more subtle individual differences in attentional responding to the threat cue.

The current study also found no support for the hypothesis; however, the results indicated that participants in the Low Worry and High Worry–Disruptive group both displayed attentional bias alignment. This demonstrates an adaptive pattern of attention allocation, as participants displayed greater attention allocation to the threat cue when it was beneficial to attend to threat, and greater attention allocation to the reward cue when it was not beneficial to attend to the threat cue. This provides support for the Dual Process Model [12] and Brandtstadter & Renner’s (1990) Theory of Assimilative and Accommodative Processes stating that the allocation of resources to goals that can be attained will be beneficial to the individual, whereas, sustained engagement with goals that cannot be attained can be detrimental to the individual. However, the finding that high disruptive worry is not associated with impaired attentional bias alignment is inconsistent with Attentional Control Theory that predicts heightened worry is associated with reduced top-down (i.e. strategic) attentional control [35].

In contrast, participants who reported that their frequent worry was non-disruptive appeared to exhibit impaired alignment of attentional bias to variations in danger controllability. Of course, caution is warranted by the marginal nature of the difference in attentional patterns between worry groups, and as the group differences in worry frequency and worry-related disruption have not been confirmed at the time of testing. However, participants in the High Worry–Non-Disruptive group seemed to display no significant differences in attentional bias to threat cues across conditions, and, instead seemed to display greater attention allocation to the reward cue in both the Control Possible and Control Not Possible blocks. This attention allocation to reward demonstrates a maladaptive pattern of attention allocation, as in the current study it is objectively more adaptive to attend to the threat cue when it was possible to avoid the noise burst and monetary loss of 15 cents, rather than attending to the reward to gain 5 cents. However, as displaying an attentional bias to threat may negatively impact emotional well-being, it may be the case that this group of participants would rather pursue the strategy of attending to the reward to sustain emotional well-being. Indeed, some research has shown that gaining a reward is more salient than avoiding a loss for some individuals [55]. It remains to be investigated whether some individuals simply attend to reward stimuli at all costs.

All participants displayed an attentional bias to reward in reward-neutral trials. In contrast in reward-threat trials, participants in the Low Worry and High Worry–Disruptive groups displayed an attentional bias to threat in Control Possible blocks. This indicates that these participants were able to alter their attentional strategy across reward-neutral and reward-threat trials types [56].

Overall, these results are consistent with theories that implicate goal-directed attentional processes in attentional biases, as the findings show that individuals can alter their attentional bias in response to contextual changes [18, 35, 57, 58]. The findings also provide support for theories suggesting that an attentional bias to threat may not be a stable phenomenon, and rather varies across time, context and threat stimuli [29, 31]. Additional results assessing participants’ response patterns to the shape identification component of the task are presented in S1 File.

Despite the possibility of the proposed hypothesis not being true, and although caution is warranted by the marginal nature of the difference between worry groups in terms of attentional bias alignment, it is also appropriate to consider possible explanations for the surprising pattern of attentional allocation in the High Worry–Non-Disruptive group. One possible explanation for this pattern of findings may be related to the group’s expectancies regarding future negative events, and subsequent learned helplessness behaviours. It may be the case that some worriers have developed negative expectancies about their control over the danger, and this has led to the belief that they have no control over the outcome of the future event. In comparison, other high worriers may have similar negative expectancies about future negative events, but their previous experiences compel them to invest cognitive and behaviour effort into attempts to control future dangers [59].

The current study may have also had methodological limitations that limited its ability to test the proposed hypothesis. Firstly, we measured worry frequency and worry-related disruption at the time of screening, and ethical constraints meant these screening data could not be matched to participants at the time of testing. As such, group averages for these screening scores could not be reported. Secondly, the measures used to assess worry frequency and worry-related disruption, may not be optimally sensitive to assessing their intended constructs. As worry is a core feature of anxiety [16], any differences between the high and low worry groups in the current study may also be attributed to group differences in anxiety. Moreover, worry-related disruption was also measured using only one item.

A further limitation of the current study relates to the psychometric properties of the dot probe paradigm. Previous research has found that the dot probe paradigm has low internal reliability [60–62]. However, this may reflect low reliability in the phenomenon of attentional bias [63]. Research has also reported that such low internal consistency is problematic for regression-based analyses, but less so for between-group comparisons [64]. Research has suggested alternative scoring methods such as attentional bias variability that may display higher reliability [29, 65, 66], however, it is unclear whether such measures assess the same attentional processes as those the traditional scoring method was designed to index [29, 63]. Further, an innovative Dual Probe Attentional Assessment Task has recently been developed [67]. The measure of attentional bias generated from this paradigm, which was designed to assess the same attentional processes as implicated in the current hypothesis, has been shown to have high internal consistency reliability [67]. As such, to address the reliability concerns, future research may benefit from testing the current hypothesis using the Dual Probe Attentional Assessment Task or alternative methods such as eye-tracking paradigms with longer viewing times [63, 68].

As our study is the first to examine individual differences in worry-linked alignment of attentional bias to variations in danger controllability and given the above limitations, it is important for future research to replicate and extend these findings using alternative measures of worry frequency and disruption. It would also be prudent to replicate these findings with different threat cues signalling different dangers, to ensure the present findings are not restricted to threat cues signalling the risk of money loss and noise burst, and instead validate the results across a variety of tasks implementing different threat cues and dangers. Future research may also be interested in assessing the duration of one’s worry, as those who experience worry that does not have utility may be characterised by worry that persists for a longer duration [69]. Future research may examine the relationship between worry and attentional bias to uncontrollable threats vs controllable threats that can either be avoided or escaped, as avoidance and escape behaviours may contribute to different worry [70]. In addition, future research may examine the current hypothesis using measures of attentional bias that are time sensitive, such as event-related potentials [71, 72], as differences in one’s ability to divert attention away from threat may also contribute to individual differences in the experience of worry.

In conclusion, the present findings do not support the hypothesis that the degree to which people experience worry as being disruptive, is an inverse function of the degree to which their attentional bias to threat cues is aligned with variation in the controllability of the dangers predicted by these threat cues. Indeed, quite the reverse pattern of findings was obtained, with participants who reported experiencing high worry that was not disruptive exhibiting the least evidence of such alignment of attentional bias to variations in danger controllability. The present study is the first, to our knowledge, that has sought to illuminate the attentional underpinnings of the distinction between disruptive and non-disruptive worry. The present study lays a conceptual and methodological foundation for future research. It is our sincere hope that fellow researchers will build on this to extend the current understanding of the distinction between disruptive and non-disruptive worry, in order to inform the development of intervention approaches that specifically target disruptive worry, to ameliorate its negative impact on individuals and society.

## Supporting information

S1 File (DOCX)

## References

[1] Diener E. Subjective well-being. The science of happiness and a proposal for a national index. Am Psychol. 2000;55(1):34–43. 11392863

[2] Ryff CD. Psychological well-being revisited: Advances in the science and practice of eudaimonia. Psychother Psychosom. 2013;83(1):10–28. 10.1159/000353263 24281296 PMC4241300

[3] Borkovec TD. The nature, functions and origins of worry. In: Davey G& Tallis F (Eds) Worrying: Perspectives on theory, assessment and treatment. 1994. p. 5–33.

[4] Gana K, Martin B, Canouet M-D. Worry and anxiety: Is there a causal relationship? Psychopathology. 2001;34(5):221–9. 10.1159/000049314 11799316

[5] Purdon C, Harrington J. Worry in Psychopathology. In: Worry and its Psychological Disorders: Theory, Assessment and Treatment. John Wiley & Sons Incorporated; 2006. p. 41–50.

[6] Bloom JR, Stewart SL, Oakley-Girvan I, Banks PJ, Shema S. Quality of life of younger breast cancer survivors: persistence of problems and sense of well-being. Psychooncology. 2012 6 1;21(6):655–65. 10.1002/pon.1965 21538677

[7] Borkovec TD, Robinson E, Pruzinsky T, DePree JA. Preliminary exploration of worry: Some characteristics and processes. Behav Res Ther. 1983;21(1):9–16. 10.1016/0005-7967(83)90121-3 6830571

[8] Behar E, Alcaine O, Zuellig AR, Borkovec TD. Screening for generalized anxiety disorder using the Penn State Worry Questionnaire: A receiver operating characteristic analysis. J Behav Ther Exp Psychiatry. 2003;34(1):25–43. 10.1016/s0005-7916(03)00004-1 12763391

[9] Ruscio AM. Delimiting the boundaries of generalized anxiety disorder: Differentiating high worriers with and without GAD. J Anxiety Disord. 2002;16(4):377–400. 10.1016/s0887-6185(02)00130-5 12213034

[10] Watkins ER. Constructive and unconstructive repetitive thought. Psychol Bull. 2008;134(2):163–206. 10.1037/0033-2909.134.2.163 18298268 PMC2672052

[11] Gladstone G, Parker G. What’s the use or worrying? Its function and its dysfunction. Aust N Z J Psychiatry. 2003;37(3):347–54. 10.1046/j.1440-1614.2003.01187.x 12780475

[12] Brandtstädter J, Rothermund K. The life-course dynamics of goal pursuit and goal adjustment: A two-process framework. Dev Rev. 2002;22(1):117–50.

[13] Leahy RL. The worry cure: Seven steps to stop worry from stopping you. New York, United States: Harmony Books; 2005.

[14] Clark DA, Beck AT. Cognitive Therapy of Anxiety Disorders: Science and Practice. New York, United States: Guilford Publications; 2011.

[15] Ruscio AM, Borkovec TD. Experience and appraisal of worry among high worriers with and without generalized anxiety disorder. Behav Res Ther. 2004;42(12):1469–82. 10.1016/j.brat.2003.10.007 15500816

[16] American Psychiatric Association. Diagnostic and Statistical Manual of Mental Disorders (DSM-5®). Washington: American Psychiatric Publishing; 2013.

[17] Hilton MF, Scuffham PA, Vecchio N, Whiteford HA. Using the interaction of mental health symptoms and treatment status to estimate lost employee productivity. Aust N Z J Psychiatry. 2010;44(2):151–61. 10.3109/00048670903393605 20113304

[18] Hirsch CR, Mathews A. A cognitive model of pathological worry. Behav Res Ther. 2012;50(10):636–46. 10.1016/j.brat.2012.06.007 22863541 PMC3444754

[19] Mathews A, MacLeod C. Cognitive vulnerability to emotional disorders. Annu Rev Clin Psychol. 2005;1(1):167–95. 10.1146/annurev.clinpsy.1.102803.143916 17716086

[20] Mathews A. Cognitive approaches to emotion and emotional disorders. Annu Rev Psychol. 1994;45(1):25–50. 10.1146/annurev.ps.45.020194.000325 8135504

[21] Oathes DJ, Squillante CM, Ray WJ, Nitschke JB. The impact of worry on attention to threat. PLoS One. 2010;5(10). 10.1371/journal.pone.0013411 20976238 PMC2954811

[22] MacLeod C, Mathews A, Tata P. Attentional bias in emotional disorders. Fowles DC (editor), editor. J Abnorm Psychol. 1986;95(1):15–20. 10.1037//0021-843x.95.1.15 3700842

[23] Bradley BP, Mogg K, White J, Groom C, De Bono J. Attentional bias for emotional faces in generalized anxiety disorder. Br J Clin Psychol. 1999 9 1;38(3):267–78. 10.1348/014466599162845 10532148

[24] Hirsch CR, MacLeod C, Mathews A, Sandher O, Siyani A, Hayes S. The contribution of attentional bias to worry: Distinguishing the roles of selective engagement and disengagement. J Anxiety Disord. 2011;25(2):272–7. 10.1016/j.janxdis.2010.09.013 20980126 PMC3034027

[25] Goodwin H, Eagleson C, Mathews A, Yiend J, Hirsch C. Automaticity of attentional bias to threat in high and low worriers. Cognit Ther Res. 2017;41(3):479–88. 10.1007/s10608-016-9818-5 28515541 PMC5410212

[26] Krebs G, Hirsch CR, Mathews A. The effect of attention modification with explicit vs. minimal instructions on worry. Behav Res Ther. 2010;48(3):251–6. 10.1016/j.brat.2009.10.009 19926075

[27] MacLeod C, Clarke PJ. The attentional bias modification approach to anxiety intervention. Clin Psychol Sci. 2015 1 1;3(1):58–78.

[28] Van Bockstaele B, Verschuere B, Tibboel H, De Houwer J, Crombez G, Koster EHW. A review of current evidence for the causal impact of attentional bias on fear and anxiety. Psychol Bull. 2014;140(3):682–721. 10.1037/a0034834 24188418

[29] Zvielli A, Bernstein A, Koster E. Temporal dynamics of attentional bias. Clin Psychol Sci Sci. 2015;3(5):772–88.

[30] Zvielli A, Bernstein A, Koster EHW. Dynamics of attentional bias to threat in anxious adults: Bias towards and/or away? PLoS One. 2014;9(8):e104025. 10.1371/journal.pone.0104025 25093664 PMC4122432

[31] Notebaert L, Georgiades JV, Herbert M, Grafton B, Parsons S, Fox E, et al. Trait anxiety and the alignment of attentional bias with controllability of danger. Psychol Res. 2018;84(3):743–56. 10.1007/s00426-018-1081-9 30132194

[32] Öhman A, Mineka S. Fears, phobias, and preparedness: Toward an evolved module of fear and fear learning. Psychol Rev. 2001;108(3):483–522. 10.1037/0033-295x.108.3.483 11488376

[33] Dolan RJ. Emotion, Cognition, and Behavior. Science (80-). 2002;298(5596):1191–4. 10.1126/science.1076358 12424363

[34] Herbert M, Notebaert L, Parsons S, Fox E, MacLeod C. The effect of varying danger controllability on attention to threat messages. Appl Cogn Psychol. 2020;34(2):425–33.

[35] Eysenck MW, Derakshan N, Santos R, Calvo MG. Anxiety and cognitive performance: Attentional control theory. Emotion. 2007;7(2):336–53. 10.1037/1528-3542.7.2.336 17516812

[36] Borkovec TD, Ray WJ. Worry: A Cognitive phenomenon intimately linked to affective, physiological, and interpersonal behavioral processes. Cognit Ther Res. 1998;22(6):561–76.

[37] Brandtstädter J, Renner G. Tenacious goal pursuit and flexible goal adjustment: Explication and age-related analysis of assimilative and accommodative strategies of coping. Psychol Aging. 1990;5(1):58–67. 10.1037//0882-7974.5.1.58 2317302

[38] Wrosch C, Scheier MF, Miller GE, Schulz R, Carver CS. Adaptive self-regulation of unattainable goals: Goal disengagement, goal re-engagement, and subjective well-being. Personal Soc Psychol Bull. 2003;29(12):1494–508. 10.1177/0146167203256921 15018681

[39] Spitzer RL, Kroenke K, Williams JB, Lowe B. A brief measure for assessing Generalised Anxiety Disorder. Arch Intern Med. 2006;166(10). 10.1001/archinte.166.10.1092 16717171

[40] Faul F, Erdfelder E, Lang A-G, Buchner A. G* Power 3: A flexible statistical power analysis program for the social, behavioral, and biomedical sciences. Behav Res Methods. 2007;39(2):175–91. 10.3758/bf03193146 17695343

[41] Löwe B, Decker O, Müller S, Brähler E, Schellberg D, Herzog W, et al. Validation and standardization of the Generalized Anxiety Disorder Screener (GAD-7) in the general population. Med Care. 2017;46(3):266–74. 10.1097/MLR.0b013e318160d093 18388841

[42] Dear BF, Titov N, Sunderland M, McMillan D, Anderson T, Lorian C, et al. Psychometric Comparison of the Generalized Anxiety Disorder Scale-7 and the Penn State Worry Questionnaire for Measuring Response during Treatment of Generalised Anxiety Disorder. Cogn Behav Ther. 2011;40(3):216–27. 10.1080/16506073.2011.582138 21770844

[43] Hobbs RJ. Noise and Vibration. Ridley J (Ed), Saf Work Butterworth-Heinemann. 1990;418–40.

[44] Koster EHW, Crombez G, Van Damme S, Verschuere B, De Houwer J. Does imminent threat capture and hold attention? Emotion. 2004;4(3):312–7. 10.1037/1528-3542.4.3.312 15456400

[45] Schneider, Eschman, Zuccolotto. E-Prime 2.0 Software Package. 2002.

[46] Notebaert L, Crombez G, Van Damme S, De Houwer J, Theeuwes J. Signals of threat do not capture, but prioritize, attention: a conditioning approach. Emotion. 2011;11(1):81–9. 10.1037/a0021286 21401228

[47] Mathews A, MacLeod C. Induced processing biases have causal effects on anxiety. Cogn Emot. 2002;16(3):331–54.

[48] Corp IBM. IBM SPSS Statistics for Windows. Armonk, NY: IBM Corp.; 2018.

[49] Cohen J. Statistical Power Analysis for the Behavioral Sciences. Florence: Routledge; 1988.

[50] Cohen J. A Power Primer. Steinberg RJ (editor), editor. Psychol Bull. 1992;112(1):155–9. 10.1037//0033-2909.112.1.155 19565683

[51] Anderson BA. Social reward shapes attentional biases. Cogn Neurosci. 2016;7(1–4):30–6. 10.1080/17588928.2015.1047823 25941868 PMC4654995

[52] D’agostino RB, Belanger A, D’Agostino Jr RB. A suggestion for using powerful and informative tests of normality. Am Stat. 1990;44(4):316–21.

[53] Williams MO, Mathews A, Hirsch CR. Verbal worry facilitates attention to threat in high-worriers. J Behav Ther Exp Psychiatry. 2014;45(1):8–14. 10.1016/j.jbtep.2013.05.006 23906509 PMC3857595

[54] Lee LO, Knight BG. Attentional bias for threat in older adults: Moderation of the positivity bias by trait anxiety and stimulus modality. Psychol Aging. 2009;24(3):741–7. 10.1037/a0016409 19739931 PMC2743240

[55] Barbaro L, Peelen M V., Hickey C. Valence, not utility, underlies reward-driven prioritization in human vision. J Neurosci. 2017;37(43):10438–50. 10.1523/JNEUROSCI.1128-17.2017 28951452 PMC6596625

[56] MacLeod C, Grafton B, Notebaert L. Anxiety-Linked Attentional Bias: Is It Reliable? Annu Rev Clin Psychol. 2019;15(1):529–54. 10.1146/annurev-clinpsy-050718-095505 30649926

[57] Eysenck MW. Anxiety: The cognitive perspective. Hove, U.K: Lawrence Erlbaum; 1992. (Essays in cognitive psychology).

[58] Cisler JM, Koster EHW. Mechanisms of attentional biases towards threat in anxiety disorders: An integrative review. Clin Psychol Rev. 2010 3;30(2):203–16. 10.1016/j.cpr.2009.11.003 20005616 PMC2814889

[59] Beck AT. Cognitive therapy and the emotional disorders. Penguin; 1976.

[60] Chapman A, Devue C, Grimshaw GM. Fleeting reliability in the dot-probe task. Psychol Res. 2019;83(2):308–20. 10.1007/s00426-017-0947-6 29159699

[61] Kappenman ES, Farrens JL, Luck SJ, Proudfit GH. Behavioral and ERP measures of attentional bias to threat in the dot-probe task: Poor reliability and lack of correlation with anxiety. Front Psychol. 2014;5(1368):1–9. 10.3389/fpsyg.2014.01368 25538644 PMC4255626

[62] Schmukle SC. Unreliability of the dot probe task. Eur J Pers. 2005;19(7):595–605.

[63] MacLeod C, Grafton B, Notebaert L. Anxiety-Linked Attentional Bias: Is it reliable? Annu Rev Clin Psychol. 2019;15(1):529–54. 10.1146/annurev-clinpsy-050718-095505 30649926

[64] De Schryver M, Hughes S, Rosseel Y, De Houwer J. Unreliable yet still replicable: A comment on lebel and paunonen (2011). Front Psychol. 2016;6(2039):1–8. 10.3389/fpsyg.2015.02039 PMC4710742 26793150

[65] Clarke PJF, Marinovic W, Todd J, Basanovic J, Chen NTM, Notebaert L. What is attention bias variability? Examining the potential roles of attention control and response time variability in its relationship with anxiety. Behav Res Ther. 2020;135:103751. 10.1016/j.brat.2020.103751 33070010

[66] Naim R, Abend R, Wald I, Eldar S, Levi O, Fruchter E, et al. Threat-related attention bias variability and posttraumatic stress. Am J Psychiatry. 2015;172(12):1242–50. 10.1176/appi.ajp.2015.14121579 26206076 PMC6335584

[67] Grafton B, Teng S, MacLeod C. Two probes and better than one: Development of a psychometrically reliable variant of the attentional probe task. Behav Res Ther. 2021;138:103805. 10.1016/j.brat.2021.103805 33485106

[68] Price RB, Silk JS, Dahl RE, Ryan ND, Kuckertz JM, Ladouceur CD, et al. Empirical recommendations for improving the stability of the dot-probe task in clinical research. Psychol Assess. 2014;27(2):365–76. 10.1037/pas0000036 25419646 PMC4442069

[69] Wells A. The Metacognitive Model of GAD: Assessment of meta-worry and relationship with DSM-IV Generalized Anxiety Disorder. Cogn Ther Res. 2005 2;29(1):107–21.

[70] Sege CT, Bradley MM, Lang PJ. Avoidance and escape: Defensive reactivity and trait anxiety. Behav Res Ther. 2018;104(843):62–8. 10.1016/j.brat.2018.03.002 29549752 PMC5903567

[71] Gaspar JM, McDonald JJ. High level of trait anxiety leads to salience-driven distraction and compensation. Psychol Sci. 2018;29(12):2020–30. 10.1177/0956797618807166 30388059

[72] Salahub C, Emrich SM. Fear not! Anxiety biases attentional enhancement of threat without impairing working memory filtering. Cogn Affect Behav Neurosci. 2020;20(6):1248–60. 10.3758/s13415-020-00831-3 32948915

