# Supplementary material for: The relationship between worry and attentional bias to threat cues signalling controllable and uncontrollable dangers
Source: PLoS One. 2021 May 13;16(5):e0251350. doi: 10.1371/journal.pone.0251350 (PMC8118555; doi:10.1371/journal.pone.0251350)
Supplement: S1 File — (DOCX) [file pone.0251350.s001.docx]

**S1 Appendix**

**Analysis of Shape Discrimination Responses**

To examine response patterns in the shapes participants selected at the end of the trial, the shape discrimination responses were also examined. S1 Table reports the average proportion of trials in which each participant group identified the opening in the reward and threat shapes in reward-neutral and reward-threat trials, for Control Possible and Control Not Possible Blocks. Note that on no trials a neutral shape was selected. Overall, participants’ shape discrimination accuracy to identify the gap in one of the shapes presented was very high (*M*  = .97, *SD* = .05).

**S1 Table. Behavioural Shape Discrimination Response in Reward-Threat trials and Reward-Neutral trials for both blocks in the Attentional Bias Alignment Assessment Task (M, SD) for the Three Participant Groups.**

|  | **Low Worry** | | **HW-ND** | | **HW-D** | |
| --- | --- | --- | --- | --- | --- | --- |
| **Reward-Threat trials** | **CP** | **CNP** | **CP** | **CNP** | **CP** | **CNP** |
| Reward shape identification | .17 (.27) | .94 (.07) | .11 (.14) | .85 (.20) | .08 (.11) | .86 (.23) |
| Threat shape identification | . 79 (.27) | .03 (.07) | .84 (.18) | .12 (.20) | .89 (.13) | .09 (.23) |
| **Reward-Neutral trials** | **CP** | **CNP** | **CP** | **CNP** | **CP** | **CNP** |
| Reward shape identification | .99 (.02) | .98 (.03) | 97 (.09) | .98 (.02) | 98 (.03) | .99 (.02) |

Note. The data presented represents the proportion of trials in which participants accurately identified the opening in the shape. Participants did not identify the neutral cue in the reward-neutral trials. HW-ND = High Worry-Non-Disruptive. HW-D = High Worry-Disruptive. CP = Control Possible. CNP = Control Not Possible.

To examine patterns in the correct discrimination of the location of the opening in the threat cue in reward-threat trials, a 2 x 3 Mixed Design ANOVA was conducted using average correct discrimination of the threat cue in reward-threat trials. The within subjects factor was Block Type (Control Possible vs Control Not Possible) and the between subjects factor was Worry Group ((Low Worry vs High Worry - Non-Disruptive vs High Worry - Disruptive). Results revealed a significant main effect of Block Type, *F*(1, 87) = 751.57, *p* <.001, *Ƞp^2^* = .90, reflecting that as expected, on average participants were more likely to correctly identify the opening in the threat cue in Control Possible blocks (*M* = .84, *SD* = .21), than in Control Not Possible blocks (*M* = .08, *SD* = .18). Results indicated a significant main effect of Worry Group *F* (2, 87) = 3.23, *p* =.04, *Ƞp^2^* = .07, indicating that the High Worry – Disruptive (*M* = .49, *SE* = .03) and High Worry – Non-Disruptive (*M* = .48, *SE* = .03) groups displayed higher threat shape discrimination accuracy, in comparison to the Low Worry group (*M* = .41, *SE* = .02). The results also revealed that the interaction between Block Type and Worry Group was non-significant, *F*(2, 87) = .53*, p* = .59.

To examine patterns in the correct discrimination of the location of the opening in the reward cue in reward-threat trials, a 2 x 3 Mixed Design ANOVA was conducted using average correct discrimination of the reward cue in reward-threat trials. The within subjects factor was Block Type (Control Possible vs Control Not Possible) and the between subjects factor was Worry Group ((Low Worry vs High Worry - Non-Disruptive vs High Worry - Disruptive). Results revealed a significant main effect of Block Type, *F*(1, 87) = 746.23, *p* <.001, *Ƞp^2^* = .90, reflecting that as expected on average participants were more likely to correctly identify the reward cue in Control Not Possible blocks (M = .88, SD = .18), than in Control Possible blocks (*M* = .12, *SD* = .19). Results indicated a significant main effect of Worry Group *F* (2, 87) = 3.60, *p* =.03, *Ƞp^2^* = .08, indicating that the High Worry – Disruptive (*M* = .47, *SE* = .03) and High Worry – Non-Disruptive (*M* = .48, *SE* = .02) groups displayed lower reward shape discrimination accuracy, in comparison to the Low Worry group (*M* = .55, *SE* = .02). The results also revealed that the interaction between Block Type and Worry Group was non-significant, *F*(2, 87) = .23*, p* = .80.

To examine patterns in the correct discrimination of the location of the opening in the reward cue in reward-neutral trials, a 2 x 3 Mixed Design ANOVA was conducted using average correct discrimination of the reward cue in reward-neutral trials, for both block types. Again, the within subjects factor was Block Type (Control Possible vs Control Not Possible) and the between subjects factor was Worry Group (Low Worry vs High Worry - Non-Disruptive vs High Worry - Disruptive). Results revealed no significant main effects of Block Type, *F*(1, 87) = .66, *p* = .42, indicating that as expected participants’ shape discrimination accuracy for reward was not different in Control Possible blocks (*M* = .98, *SD* = .06) and Control Not Possible blocks (*M* = .98, *SD* = .02). Results also revealed no significant main effect of Worry Group *F* (2, 87) = .21, *p* =.81, and the interaction between Block Type and Worry Group was also non-significant, *F* (2, 87) = 1.90, *p* =.16.

In summary, analysis of the shape discrimination responses indicated that there is dissociation between participants’ pattern of attentional bias to threat and the shape discrimination response. Specifically, all three Worry Groups as expected identified the opening in the threat cue more in Control Possible blocks, than in Control Not Possible blocks, and the opening in the reward cue more in Control Not Possible blocks, than in Control Possible blocks. Although overall the two high worry groups appeared more likely to identify the opening in the threat shape, than in the reward shape, as compared to the low worry group, this pattern was not further modified by block type. As such, even though participants in the High Worry-Non-Disruptive group uniquely did not show an attentional bias to the threat cue in Control Possible Blocks, they did not differ from participants in the High Worry-Disruptive group in their accuracy to identify the opening in the threat cue. This suggests a dissociation between the attention and behavioural responses in the High Worry-Non-Disruptive group. Such a dissociation could arise either because the observed pattern of attentional bias is the result of early automatic processes, which was later overwritten to produce the behavioural response, or because the observed pattern of attentional bias is the result of strategic processes, which have overwritten earlier automatic attentional processes. Future research could examine whether the observed pattern of attentional bias is the result of automatic or strategic processes, for example by manipulating stimulus exposure duration.
